# Gender Differences in Work Status during Early Career of Dentists: An Analysis of National Survey Cohort Data of 10 Years in Japan

**DOI:** 10.3390/ijerph18052335

**Published:** 2021-02-27

**Authors:** Katsuo Oshima, Tomoko Kodama, Yusuke Ida, Hiroko Miura

**Affiliations:** 1 Department of Dental Technology, The Nippon Dental University College, Tokyo 102-8159, Japan; 2 Department of International Health and Collaboration, National Institute of Public Health, Saitama 351-0197, Japan; kodama.t.aa@niph.go.jp; 3 Healthcare Executive Program, Graduate School of Medicine, The University of Tokyo, Tokyo 113-8655, Japan; yskida@m.u-tokyo.ac.jp; 4 Division of Disease Control and Epidemiology, School of Dentistry, Health Sciences University of Hokkaido, Ishikari 061-0293, Japan; hmiura@hoku-iryo-u.ac.jp

**Keywords:** gender differences, career paths, work status, female dentists, career break, return to work, Japan

## Abstract

Few studies have evaluated gender differences in young dentists’ career focusing on career breaks and return to work. We created a cohort dataset for dentists registered in 2006 using the national survey between 2006–2016 (men, 1680; women, 984), and examined the work setting of dentists by gender 10 years after registration. The proportion of dentists on career break increased each survey year, and was more pronounced in women than in men (2006 to 2016, men, 11.2% to 14.2%; women, 7.9% to 31.0%). The proportion of those who had career breaks between 2006–2016 was 44.8% in men and 62.9% in women. In the multiple logistic regression for examining the associations between those who returned to work compared to those working continuously, in women, the odds ratios (OR) were significantly higher in those working in dental clinics (owner, OR: 5.39; employee, OR: 3.10), and those working part-time (OR: 2.07); however, in men, there was no significant association with part-time work. These results suggest during early career phase, female dentists are more likely than males to take career breaks and choose part-time on returning. These gender differences should be considered for ensuring adequate workforce in dentistry in the future.

## 1. Introduction

An increasing proportion of female graduates from dental schools has been reported in many countries [1,2,3,4]. Tiwari et al. [4] investigated the gender distribution of dental school graduates at an international level. In North America, the proportions of male and female graduates in 2018 were almost equal [1,4]. In Europe, the proportion of female dental graduates varied, and in 10 European countries at least 70% of the graduates were women in 2016 [2,4]. In Japan, the proportion of female graduates was 41% in 2014 [3,4]; though this is comparatively low, the number of female graduates has been increasing in the recent years [5]. These reports show a global trend of growing female workforce in dentistry, which may further rise in the future.

Nevertheless, several reports have suggested that compared to male dentists, female dentists face many issues in dental practice, such as those related to working hours and career breaks [6,7,8,9,10,11]. Studies in various countries have reported that working hours of female dentists in dental practice are shorter than male dentists [7,8,9,10,11]. Similarly, it was found that female dentists take career breaks more often than male dentists [6,8]. A questionnaire survey by the General Dental Council in UK found that 27% of male dentists and 61% of female dentists had taken a career break at some point in their lives [6]. In a similar survey of dentists in New Zealand, it was reported that 28.6% of male dentists and 62.6% of female dentists had taken a career break [8], supporting the findings of the study conducted in UK. It has been suggested that the major reason for short working hours and leave of absence of female dentists is childcare [8,10,11]. Moreover, young dentists often face many hurdles at work and in their family-life while their career is developing [9]; particularly, women are more likely to feel insecure about their future prospects [12], and to suffer burnout [13].

These previous studies suggest that especially during the early stages in their career, female dentists have many barriers in dental practice than male dentists, and they may sometimes choose to take leave. However, few studies have focused on gender differences in the transitional phases of young dentists’ career such as decision on their workplace, workstyles, career breaks, and return to work. Considering the possibility of rising number of female dentists, the workstyle and choices of female dentists in their 20’s and 30’s will be a major deciding factor for maintaining dental workforce in the future. Therefore, to improve the work environment of female dentists, policy interventions are needed, for which it is necessary to obtain substantial recent information on a national scale. 

We obtained data from the National Survey of Physicians, Dentists, and Pharmacists (NSPDP) 2006–2016 conducted by the Ministry of Health, Labour and Welfare (MHLW) of Japan, and then generated 10 years cohort dataset using individual data of dentists registered in 2006. The NSPDP is a mandatory survey conducted every 2 years with the aim of understanding the work status of Japanese dentists on a national scale [14]. Thus, the purpose of our study was to examine the transition of the work setting of the dentists registered in 2006 according to gender during the 10-year period from 2006 to 2016, using the cohort dataset. We also assessed whether the participants took career breaks between 2006 and 2016. Finally, among those who were working as of 2016, we evaluated the association between those who returned to work after a career break, if ever taken, and their characteristics such as gender, age, work setting, workplaces, and working hours.

## 2. Materials and Methods

### 2.1. Data Acquisition and Preparation for Analysis

In Japan, pursuant to the Dental Practitioners’ Law, all dentists are required to report their current work status to the MHLW once every 2 years through their prefecture, regardless of whether the dentist is actually practicing. Based on the Statistics’ Law, we obtained individual data from the NSPDP, with official permission from the MHLW, for 2006, 2008, 2010, 2012, 2014, and 2016. The data included gender, age, registration number, registration year, types of work setting, working hours (this item was used for the 2016 survey only), and municipal code of workplace of dentists. Dentist names and their facility names were not collected to protect their privacy.

**Figure 1:**
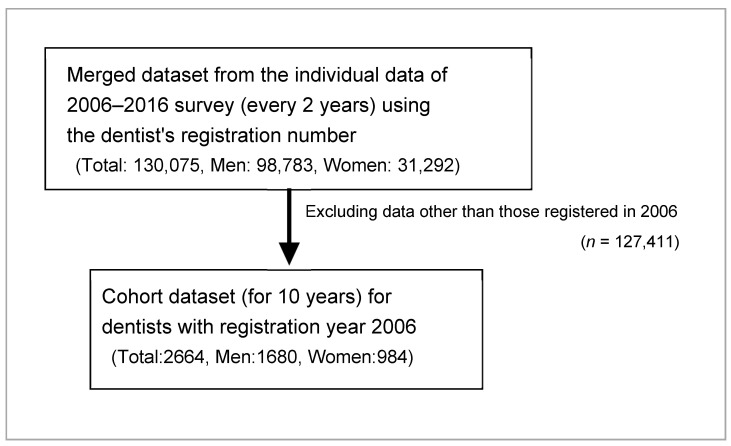
Method of acquisition and preparation of data of the study participants.

To prepare for analysis, we first merged all the data of each dentist for each year from 2006 to 2016 using the dentist’s registration number (total, 130,075 dentists; men, 98,783; women, 31,292). From this, we deleted the data of all dentists except those who became dentists/first registered as dentists in 2006, and created a cohort dataset for dentists with registration year 2006 (total, 2664 dentists; men, 1680; women, 984) (Figure 1).

This cohort dataset contains some unreported dentists depending on the year, in spite of the fact that participation in the survey is mandatory for all. In such cases, they were considered to be engaged in work unrelated to dental health care, because the survey is sent to workplace; therefore, we defined that the dentist was on leave [15,16]. With regard to the dentist’s gender and age, we used data reported by the same dentist in surveys of other years and filled in all required information. It was assumed that there were no deceased subjects. In this study, we analyzed the cohort data of dentists registered in 2006. In total, 2673 individuals passed the 2006 National Dental Examination (men, 1682; women, 991) [17], and 99.7% of dentists reported to the MHLW at least once between 2006 and 2016, and they were included in this study.

### 2.2. Data Analysis

First, for each sample year (2006, 2008, 2010, 2012, 2014, 2016), we calculated the number of dentists by gender according to work environment (types of work settings, types of municipalities, and working hours). Work setting was classified as: (1) dental clinics (owner); (2) dental clinics (employee); (3) hospital (general hospitals and university hospitals); (4) other facilities (those who are enrolled in facilities other than (1)–(3) described above, such as research work, postgraduate student, nursing facilities, and administrative bodies); and (5) on career break (those who were on leave/unreported, as described above, or those who reported no employment). Municipalities was classified as: (1) metropolises (ordinance-designated cities with a population of 500,000 or more, and 23 special wards of Tokyo), (2) cities (cities with a population of 50,000 or more, excluding metropolises), and (3) towns and villages (small municipalities that do not meet city requirements). Working hours were classified as follows: (1) full-time (≥32 h/week) and (2) part-time (<32 h/week). We then graphically illustrated the transition of the types of dentists’ work settings during the 10 years since registration as dentists (i.e., from 2006 to 2016), and compared gender differences in work setting using chi-square test.

Second, we assessed whether the participants had a career break between 2006 and 2016. If there is one or more career break during the last 10 years, it was defined as “ever to have taken a career break”. Based on these results, we divided the participants into three categories as follows: those who never took a career break and continuously practiced dentistry between 2006 and 2016 was the “continuous practice” group; those who had ever taken a career break but practicing as of 2016 was classified in the “returned to practice” group; those who were on career break as of 2016 was classified in the “on career break” group. We analyzed each group based on their proportion of each gender and evaluated their relationship to the characteristics of the participants as of 2016. Chi-square test was used to compare the proportion of each data.

To examine the characteristics of “returned to practice” (i.e., returned to practice group vs. continuous practice group), a multiple logistic regression analysis was conducted. Data on “returned to practice” group (men, 514; women, 314) and “continuous practice” group (men, 928; women, 365) were included in the analysis. To analyze the primary outcome variable of “returned to practice” group, explanatory variables of gender (men/women), age (34–36, 37–39, 40–42, and ≥43 years), municipalities (metropolises, cities, and towns and villages), and working hours (full-time/part-time) were explored. This analysis was also performed on each gender. The odds ratio (OR) and the 95% confidence interval (95% CI) of each explanatory variable were calculated in a forced entry model. For data management and statistical analyses in this study, STATA version 14 (StataCorp LLC, College Station, TX, USA) was used. *p* values < 0.05 was regarded as significant.

### 2.3. Ethical Consideration

The individual data from the NSPDP were electronic with no identifying information, except for each dentist’s registration number, which was not disclosed to the public and could not be linked to the dentists’ private information, such as name and address. The data were used in accordance with the Statistics’ Law with consideration for protecting confidentiality and ensuring proper management. This study was approved by the Research Ethics Committee of the National Institution of Public Health in September 2019 (NIPH-IBRA #12250). The requirement for consent from participants was waived as this study was a secondary data analysis of a government survey.

## 3. Results

### 3.1. Changes in Work Status of the Study Participants

**Figure 2:**
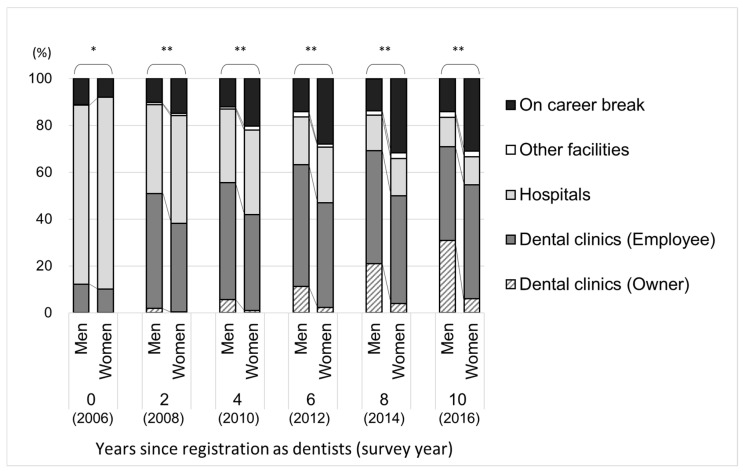
Proportion of work setting types of the participants in each survey year (men: 1680, women: 984). Chi-square test: * *p* < 0.01, ** *p* < 0.001.

Table 1 presents the number and proportions of study participants according to age, types of work setting, municipalities, and working hours, in the first year of registration as dentists (2006) and 10 years later (2016). The changes in the proportion of types of work setting at initial registration and after the follow-up period of 10 years, respectively, are presented in Figure 2. The number and proportion of the above data in each year between 2006 and 2016 are presented in Supplementary Table S1. When comparing the proportion of types of work setting between men and women in 2006, their work status tendencies were similar; however, after 10 years (2016), men and women showed different tendencies.

Between 2006 and 2016, among male dentists, while the proportion of those working at hospitals gradually decreased, the proportion of dentists working in dental clinics increased, especially the proportion of dental clinic owners. Among male dentists, in 2016, dental clinics (as employees) was the most frequent work setting (40.0%), followed by dental clinics (as owners; 30.8%), and hospitals (12.6%). Among female dentists, while the proportion of those working at hospitals gradually decreased, the proportion of dentists working in dental clinics increased, but only slight increase was seen in the proportion of dental clinic owners. Among female dentists, dental clinics (as employee) was the most common work setting (48.6%), followed by academic institutions (12.0%), and dental clinics (as owners; 6.0%).

For both men and women, the proportion of those on career break gradually increased from 2006 to 2016. This tendency was more pronounced in female dentists than in male dentists (from 2006 to 2016: men: 11.2% to 14.2%, women: 7.9 to 31.0%).

### 3.2. Gender Differences in Proportion of Those Who Ever Had a Career Break between 2006 and 2016

**Figure 3:**
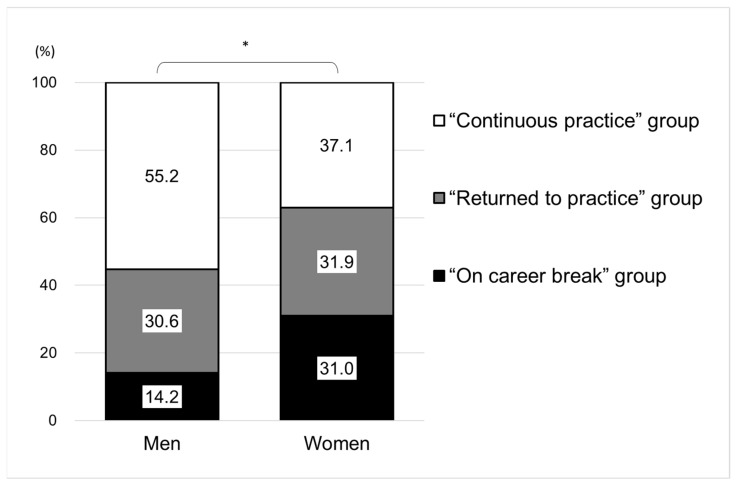
Proportion of three categories by gender (men: 1680, women: 984). “Continuous practice” group: those who were working continuously between 2006 and 2016, “Returned to practice” group: those who ever had a career break but working as of 2016, “On career break” group: those who were on career break as of 2016. Chi-square test: * *p* < 0.001.

Figure 3 shows a comparison by gender of whether the subjects had a career break between 2006 and 2016. Among the 1680 male dentists, 55.2% belong to continuous practice group, while 30.6% in the returned to practice group, and 14.2% was on career break. Among the 984 female dentists, 37.1% was in the continuous practice group, 31.9% in the returned to practice group, and 31.0% was on career break (*p* < 0.001). The proportion of those who ever had a career break between 2006 and 2016 was 44.8% in men and 62.9% in women.

Table 2 shows the relationship between each category and the characteristics of the participants as of 2016. Among men, there were significant differences between the two groups (continuous/returned to practice) in “age category (*p* = 0.041)” and “work setting (*p* = 0.003)”. With regard to the work setting of men, those who returned to practice was higher in dental clinics and lower in hospitals. Among women, there were significant differences between the two groups (continuous/returned to practice) in “work setting (*p* < 0.001)” and “working hours (*p* < 0.001)”. Higher number of female dentists working in dental clinics returned to practice compared to those working in hospitals, and were more likely to work part-time.

### 3.3. Characteristics Associated with the Dentists Who Returned to Practice as of 2016

Table 3 presents the odds ratios in multiple logistic regression analysis for the returned to practice group compared to continuous practice group, adjusted for their relationship to the participants characteristics described in Table 2. 

In the analysis of male dentists, the odds ratios were significantly higher in those aged 40–42 years (OR: 1.60, 95%CI: 1.06–2.41), and those working in dental clinics (owner, OR: 1.94, 95%CI: 1.34–2.80; employee, OR: 1.71, 95%CI: 1.20–2.43) and other facilities (OR: 2.18, 95%CI: 1.08–4.43). In the analysis of female dentists, the odds ratios were significantly higher in those working in dental clinics (owner, OR: 5.39, 95%CI: 2.69–10.80; employee, OR: 3.10, 95%CI: 1.93–4.99), and those who working part-time (OR: 2.07, 95%CI: 1.48–2.88).

## 4. Discussion

### 4.1. Main Findings

In this study, we evaluated gender differences in young dentists’ career paths with focusing on career break and return to work during 10 years after registration as dentists on a national scale. Our analysis has shown that, in each survey year, the proportion of dentists on career break gradually increased, which was more pronounced in women than in men. Among the female dentists who returned to work, the odds ratios were significantly higher in those working in dental clinics, and part-time practice, after adjusting for other factors; however, in men, there was no significant association with part-time practice. Therefore, our results suggest that in the first 10 years of career as dentists, women are more likely than men to take career breaks and to work part-time after returning to practice. 

### 4.2. Gender Differences in Career Break

Previous studies have shown that women take more career breaks than men [6,8]. A UK study in 2001 reported that 61% of female dentists had taken a career break at some point in their lives, whereas it is only 27% among male dentists [6]. A study in New Zealand in 2008 found that 28.6% of male dentists and 62.6% of female dentists had taken a career break [8]. Our study showed that on a national scale, female dentists are more likely than male dentists to take a break in the initial 10 years of their dental career (men, 44.8%; women, 62.9%). Our study cannot be compared to previous studies solely based on percentage of career break differences in the analysis methods, the age group studied, and the dental health service systems in different countries; however, the fact that female dentists take more career breaks than male dentists is a common issue. 

However, in this study, it was not possible to clarify why the participants had taken career breaks. Ayers et al. [8] reported that the major reason for career break was childcare (69.9%) in female dentists, but personal choice (33.8%) and seeking job in dentistry (33.8%) in male dentists. According to a Japanese study using the NSPDP, there has been an increasing trend of unreported (it was defined as career break in this study) peaks at 25–34 years of age, and women tended to have more unreported peaks than men [15]. These suggest the likely reasons for not reporting, such career break due to childbirth/childcare and/or transfer. As most of our participants were in their 20’s as of 2006 (men, 87.6%; women, 93.8%) it is assumed that the reason for career breaks could align with the scenario stated above. 

### 4.3. Young Dentists Returning to Dental Practice

The results of this study demonstrate that when returning to work, female dentists are more likely to work in dental clinics and to choose part-time work. However, male dentists are more likely to work in dental clinics and other facilities, but only a few choose to work part-time. Several previous studies have suggested that women tend to choose part-time work, the major reason for this being childcare [8,11,18]. Additionally, it was reported that one of the reasons young dentists choose to work in dental clinic was the flexibility of work schedule [19]. A report from Japan suggests that young dentists working in hospitals have long working hours and inadequate environment to support childcare [20]. Furthermore, a questionnaire survey in Japan also reported that the working hours of female dentists employed in dental clinics are shorter than those who are owners of dental clinics [21]. Hence, considering both the results of our study and previous studies, it may be noted that female dentists owning a dental clinic are more likely to have more flexible hours. 

However, our study showed that women were less likely to become the owners of dental clinics than men, as of 2016, regardless of whether they had taken a career break. In Japan, the proportion of dentists working in dental clinics by gender was 67.0% for men (owners, 51.7%; employees, 15.3%) and 18.3% for women (owners, 5.2%, employees, 13.1%) according to the 2016 NSPDP report released by the MHLW [14]. In other countries as well, it has been reported that women were rarely the owners of dental clinics, compared to men [10,22]. In this study, as we did not analyze the reasons preventing female dentists to become clinic owners, more detailed study is needed. 

Additionally, men were more likely to return to work not only at dental clinics but also at other facilities. However, women returning to work at other facilities is relatively low. The other facilities include a large number of dentists engaged in research work. Gender differences among dentists are observed not only in clinical practice but also in academic research [4]. The results of our study also suggest gender differences in research work.

### 4.4. Relationship between Geographical Distribution and Young Dentists Returning to Practice

This study showed that, for both male and female dentists, there were no significant differences in the relationship between working status and workplace. These results suggest that there are no regional differences to dentists returning work. Although female dentists are more likely to work in urban areas [10,23], few reports have shown a relationship between regional differences in career break/return to work and gender. Reports have indicated that in Japan, the number of dentists is increasing, and their regional inequalities are declining, causing geographical diffusion [24,25]. Therefore, it is assumed that there is little regional inequality in the employment of young dentists, and similarly, there was no regional influence on the different genders returning to work. 

### 4.5. Increasing Proportion of Female Dentists and the Implications of This Study

Our study results suggest important implications on the nature of work among female dentists. Based on the relationship between gender and work settings of young dentists in this study, it may be suggested that there are various barriers in the working environment of female dentists on a national scale. 

The proportion of Japanese female dentists under 29 years of age increased dramatically from 28.2% in 1996 to 40.6% in 2006 and 44.7% in 2016 [14], and this suggests that the proportion of total female dentists would continue to rise in the future [26]. Therefore, the fact that young female dentists work fewer hours due to career break than male dentists could have an impact on the workforce in dentistry in the future. Consequently, these conditions may lead to a decline of the availability of the dental health care in the whole country. Hence, policymakers should be aware of the working conditions of female dentists and consider implementing policies to support female dentists’ working environments so that they can work at par with male dentists. 

### 4.6. Limitations of This Study

This study has several limitations. First, in this study, we divided three groups (“continuous practice”, “returned to practice”, and “on career break”) based on whether the participants had a career break between 2006 and 2016, but it is possible that “continuous practice” included those who had career breaks. This is because the NSPDP is a mandatory survey conducted every two years and if a dentist takes a career break in a year when the survey was not conducted, it is impossible to know the working status. Additionally, we did not consider the duration of the participants’ career break in our analysis because of the characteristics of the NSPDP survey period. Second, the NSPDP aims to understand the current work setting of dentists; however, it is impossible to know individual reasons for change in work setting, career break, and return to practice. Similarly, using the data in the NSPDP, it is impossible to grasp the various factors related to work setting, such as income, and satisfaction level. More data is needed to analyze the underlying reasons for the changes in the current work setting of dentists. Third, this study examined the work setting of young dentists using the cohort data of dentists registered in 2006 and did not analyze the work setting of dentists registered in other years. Further studies are required to examine potential differences between each registration year. 

Despite these limitations, to the best of our knowledge, this study is the first demonstration of gender-based differences in the career paths of dentists after registration on a national scale. The results of this study indicate that there are gender barriers to the work styles of dentists, similar to studies in several other countries [6,7,8,9,10,11], and these issues could have an impact on the dental workforce in the future.

## 5. Conclusions

We analyzed gender-based differences in young dentists’ career paths, in terms of the work status such as career break and return to practice in 10 years after registration as dentists on a national scale. We found that, in each survey year, the proportion of dentists on career break gradually increased, and this tendency was more pronounced in women than in men. The proportion of those who ever had a career break between 2006 and 2016 was 44.8% in men and 62.9% in women. Finally, with regard to the characteristics of female dentists who returned to practice, the odds ratios were significantly higher in those working in dental clinics, and those working part-time, after adjusting for other factors; however, in men, it was not significant for part-time work. In the first 10 years of their career as dentists, women are more likely than men to go on career breaks and to select part-time work after returning to practice. These issues could have an impact on the workforce in dentistry in the future because of the rising proportion of young female dentists.

## Figures and Tables

**Table 1:** Characteristics of the study participants (as of registration (2006) and 10 years later (2016)).

Variable	As of Registration (2006)				At 10 Years after Registration (2016)			
	Men (*n* = 1680)		Women (*n* = 984)		Men (*n* = 1680)		Women (*n* = 984)	
Age, mean (standard deviation)	26.9	(2.7)	26.1	(2.3)				
Age category, *n* (%)								
24–26	988	(58.8)	738	(75.0)				
27–29	484	(28.8)	185	(18.8)				
30–32	122	(7.3)	35	(3.6)				
≥33	86	(5.1)	26	(2.6)				
Work setting, *n* (%)								
Dental clinic (Owner)	0	(0.0)	0	(0.0)	518	(30.8)	59	(6.0)
Dental clinic (Employee)	206	(12.3)	99	(10.1)	672	(40.0)	478	(48.6)
Hospitals	1284	(76.4)	807	(82.0)	212	(12.6)	118	(12.0)
Other facilities	2	(0.1)	0	(0.0)	40	(2.4)	24	(2.4)
On career break	188	(11.2)	78	(7.9)	238	(14.2)	305	(31.0)
Municipalities, *n* (%)								
Metropolis (pop 500,000+)	741	(49.7)	502	(55.4)	583	(40.4)	303	(44.6)
Cities (pop 50,000+)	703	(47.1)	387	(42.7)	781	(54.2)	345	(50.8)
Towns and villages	48	(3.2)	17	(1.9)	78	(5.4)	31	(4.6)
Working hours, *n* (%)								
Full-time (≥32 h/week)					1301	(91.8)	399	(59.6)
Part-time (<32 h/week)					117	(8.2)	270	(40.4)

The age of participants given in the table is of 2006 (the age of the participants in the 2016 survey is 10 years more than the age in the 2006 survey). “Working hours” data was obtained in the 2016 survey only, and the proportion was calculated excluding missing values.

**Table 2:** Relationship between each group and the characteristics of the study participants.

Variable	Men							Women						
	Continuous Practice Group (*n* = 928)		Returned to Practice Group (*n* = 514)		On Career Break Group (*n* = 238)		*p* Value	Continuous Practice Group (*n* = 365)		Returned to Practice Group (*n* = 314)		On Career Break Group (*n* = 305)		*p* Value
	*n*	(%)	*n*	(%)	*n*	(%)		*n*	(%)	*n*	(%)	*n*	(%)	
Age category														
34–36	575	(62.0)	285	(55.4)	128	(53.8)	0.041 /0.060	284	(77.8)	244	(77.7)	210	(68.8)	0.532 /0.090
37–39	251	(27.0)	156	(30.4)	77	(32.4)		58	(15.9)	55	(17.5)	72	(23.6)	
40–42	57	(6.1)	48	(9.3)	17	(7.1)		15	(4.1)	7	(2.2)	13	(4.3)	
≥43	45	(4.9)	25	(4.9)	16	(6.7)		8	(2.2)	8	(2.6)	10	(3.3)	
Work setting														
Dental clinics (owner)	315	(33.9)	203	(39.5)			0.003	25	(6.8)	34	(10.8)			<0.001
Dental clinics (employee)	431	(46.4)	241	(46.9)				234	(64.1)	244	(77.7)			
Hospitals	159	(17.1)	53	(10.3)				90	(24.7)	28	(8.9)			
Other facilities	23	(2.5)	17	(3.3)				16	(4.4)	8	(2.6)			
Municipalities														
Metropolis (pop 500,000+)	363	(39.1)	220	(42.8)			0.200	157	(43.0)	146	(46.5)			0.477
Cities (pop 50,000+)	509	(54.8)	272	(52.9)				193	(52.9)	152	(48.4)			
Towns and villages	56	(6.0)	22	(4.3)				15	(4.1)	16	(5.1)			
Working hours														
Full-time (≥32 h/week)	845	(91.9)	456	(91.4)			0.712	244	(67.8)	155	(50.2)			<0.001
Part-time (<32 h/week)	74	(8.1)	43	(8.6)				116	(32.2)	154	(49.8)			

Regarding *p* value of “Age”, the upper value is the comparison between two groups (continuous and returned to practice), and the lower value is the comparison between three groups (continuous practice, returned to practice, and on career break).

**Table 3:** Odds ratio for the “returned to practice” group at 10 years after registration as dentists.

Variable	All (*n* = 2121)			Men (*n* = 1442)			Women (*n* = 679)		
	OR	95%CI	*p* Value	OR	95%CI	*p* Value	OR	95%CI	*p* Value
Gender									
Men	1.00	Reference							
Women	1.47	(1.19–1.81)	<0.001						
Age category									
34–36	1.00	Reference		1.00	Reference		1.00	Reference	
37–39	1.19	0.96–1.47	0.116	1.24	(0.96–1.58)	0.095	1.10	(0.72–1.68)	0.667
40–42	1.27	0.87–1.85	0.216	1.60	(1.06–2.41)	0.027	0.53	(0.20–1.39)	0.196
≥43	1.03	0.65–1.62	0.910	1.05	(0.63–1.75)	0.862	0.97	(0.34–2.77)	0.959
Work setting									
Dental clinics (Owner)	2.65	(1.93–3.62)	<0.001	1.94	(1.34–2.80)	<0.001	5.39	(2.69–10.80)	<0.001
Dental clinics (Employee)	2.22	(1.67–2.94)	<0.001	1.71	(1.20–2.43)	0.003	3.10	(1.93–4.99)	<0.001
Hospitals	1.00	Reference		1.00	Reference		1.00	Reference	
Other facilities	2.06	(1.16–3.64)	0.013	2.18	(1.08–4.43)	0.031	1.88	(0.72–4.95)	0.200
Municipalities									
Metropolis (pop 500,000+)	1.22	(1.01–1.47)	0.039	1.17	(0.93–1.48)	0.167	1.33	(0.96–1.85)	0.086
Cities (pop 50,000+)	1.00	Reference		1.00	Reference		1.00	Reference	
Towns and villages	0.89	(0.58–1.35)	0.573	0.72	(0.43–1.22)	0.223	1.26	(0.59–2.69)	0.548
Working hours									
Full-time (≥32 h/week)	1.00	Reference		1.00	Reference		1.00	Reference	
Part-time (<32 h/week)	1.64	(1.28–2.11)	<0.001	1.15	(0.76–1.73)	0.503	2.07	(1.48–2.88)	<0.001

OR: odds ratio, 95%CI: 95% confidence interval.

## Data Availability

The raw data collected for government statistics cannot be shared because of restrictions stipulated by the Ministry of Health, Labour and Welfare.

## Supplementary Materials

The following are available online at https://www.mdpi.com/1660-4601/18/5/2335/s1, Table S1: The number and proportion of participants between 2006 and 2016.

## References

[1] American Dental Association (2018). 2017–2018 Survey of Dental Education—Report 1: Academic Programs, Enrollment, and Graduates.

[2] Kravitz A., Bullock A., Cowpe J., Barnes M. (2016). Manual of Dental Practice 2015.

[3] Japan Dental Association (2015). Basic Information on Dental and Oral Health. https://www.jda.or.jp/dental_data/pdf/chapter_04.pdf.

[4] Tiwari T.R., Randall C.L., Cohen L., Holtzmann J., Webster-Cyriaque J., Ajiboye S.A., Schou L., Wandera M., Ikeda K., Fidela de Lima Navarro M. (2019). Gender inequalities in the dental workforce: Global perspectives. Adv. Dent. Res..

[5] Ono K. (2017). Admission of dental students. J. Jpn. Dent. Educ. Assoc..

[6] Newton J.T., Buck D., Gibbons D.E. (2001). Workforce planning in dentistry: The impact of shorter and more varied career patterns. Community Dent. Health.

[7] del Aguila M.A., Leggott P.J., Robertson P.B., Porterfield D.L., Felber G.D. (2005). Practice patterns among male and female general dentists in a Washington State population. J. Am. Dent. Assoc..

[8] Ayers K.M., Thomson W.M., Rich A.M., Newton J.T. (2008). Gender differences in dentists’ working practices and job satisfaction. J. Dent..

[9] Pallavi S.K., Rajkumar G.C. (2011). Professional practice among woman dentist. J. Int. Soc. Prev. Community Dent..

[10] McKay J.C., Quiñonez C.R. (2012). The feminization of dentistry: Implications for the profession. J. Can. Dent. Assoc..

[11] McKay J.C., Ahmad A., Shaw J.L., Rashid F., Clancy A., David C., Figueiredo R.L.F., Quiñonez C. (2016). Gender differences and predictors of work hours in a sample of Ontario dentists. J. Can. Dent. Assoc..

[12] Campus G., Rusca P., Amrhein C., Meier A., Zeyer O., Wolf T.G. (2020). Career Prospects of Young Dentists in Switzerland. Int. J. Environ. Res. Pub. Health.

[13] Kulkarni S., Dagli N., Duraiswamy P., Desai H., Vyas H., Baroudi K. (2016). Stress and professional burnout among newly graduated dentists. J. Int. Soc. Prev. Community Dent..

[14] Ministry of Health, Labour and Welfare Survey of Physicians, Dentists, and Pharmacists. https://www.mhlw.go.jp/toukei/list/33-20_old.html.

[15] Shimada N., Kondo T. (2004). Estimation of actual report rates using data from the survey of physicians, dentists and pharmacists. Nihon Koshu Eisei Zasshi.

[16] Kodama T., Koike S., Matsumoto S., Ide H., Yasunaga H., Imamura T. (2012). The working status of Japanese female physicians by area of practice: Cohort analysis of taking leave, returning to work, and changing specialties from 1984 to 2004. Health Policy.

[17] Ministry of Health, Labour and Welfare Current Status of Clinical Training for Dentists. https://www.mhlw.go.jp/stf/seisakunitsuite/bunya/0000085959.html.

[18] Kuthy R.A., Jennings A.D., McQuistan M.R., Marshall T.A., Qian F. (2013). Influence of minor children and contribution to household income on work hours of female dentists. J. Public Health Dent..

[19] Gallagher J.E., Clarke W., Eaton K.A., Wilson N.H. (2007). Dentistry—A professional contained career in healthcare. A qualitative study of Vocational Dental Practitioners’ professional expectations. BMC Oral Health.

[20] Kodama T., Ida Y., Miura H.A. (2020). Nationwide Survey on Working Hours and Working Environment among Hospital Dentists in Japan. Int. J. Environ. Res. Public Health.

[21] Karube H., Suetaka T. (2006). Operation situation of female dentists of about 50 years of age. Jpn. J. Dent. Prac. Admin..

[22] Riley J.L., Gordan V.V., Rouisse K.M., McClelland J., Gilbert G.H. (2011). Dental Practice-Based Research Network Collaborative Group. Differences in male and female dentists’ practice patterns regarding diagnosis and treatment of dental caries: Findings from The Dental Practice-Based Research Network. J. Am. Dent. Assoc..

[23] McCarthy G.M., MacDonald J.K. (1996). Gender differences in characteristics, infection control practices, knowledge and attitudes related to HIV among Ontario dentists. Community Dent. Oral. Epidemiol..

[24] Toyokawa S., Kobayashi Y. (2010). Increasing supply of dentists induces their geographic diffusion in contrast with physicians in Japan. Soc. Sci. Med..

[25] Morita T., Hashimura T., Senoo Y., Tanimoto T. (2019). Trend in unequal geographical distribution of dentists by age and gender in Japan from 1996—2014. Community Dent. Health.

[26] Ishimaru M., Ono S., Yasunaga H., Matsui H., Koike S. (2016). Projected future distribution of dentists in Japan. J. Public Health Dent..

