# Supplementary material for: Gender Differences in Work Status during Early Career of Dentists: An Analysis of National Survey Cohort Data of 10 Years in Japan
Source: Int J Environ Res Public Health. 2021 Feb 27;18(5):2335. doi: 10.3390/ijerph18052335 (PMC7967721; doi:10.3390/ijerph18052335)
Supplement: Supplementary file 1 [file ijerph-18-02335-s001.pdf]

Supplementary Table S1. The number and proportion of the participants between 2006 and 2016

	As of registration (2006)				After 2 years (2008)				After 4 years (2010)				After 6 years (2012)				After 8 years (2014)				After 10 years (2016)			
	Men		Women		Men		Women		Men		Women		Men		Women		Men		Women		Men		Women	
Work setting, n (%)																								
Dental clinics (Owner)	0	(0.0)	0	(0.0)	33	(2.0)	3	(0.3)	94	(5.6)	9	(0.9)	190	(11.3)	23	(2.3)	352	(21.0)	38	(3.9)	518	(30.8)	59	(6.0)
Dental clinics (Employee)	206	(12.3)	99	(10.1)	822	(48.9)	372	(37.8)	838	(49.9)	403	(41.0)	872	(51.9)	439	(44.6)	811	(48.2)	454	(46.1)	672	(40.0)	478	(48.6)
Hospitals	1,284	(76.4)	807	(82.0)	639	(38.0)	453	(46.0)	530	(31.6)	355	(36.1)	343	(20.4)	234	(23.8)	255	(15.2)	155	(15.8)	212	(12.6)	118	(12.0)
Others	2	(0.1)	0	(0.0)	13	(0.8)	10	(1.0)	14	(0.8)	17	(1.7)	36	(2.2)	12	(1.2)	32	(1.9)	25	(2.5)	40	(2.4)	24	(2.4)
On career break	188	(11.2)	78	(7.9)	173	(10.3)	146	(14.9)	204	(12.1)	200	(20.3)	239	(14.2)	276	(28.1)	230	(13.7)	312	(31.7)	238	(14.2)	305	(31.0)
Municipality, n (%)																								
Metropolis (pop 500,000+)	741	(49.7)	502	(55.4)	718	(47.6)	417	(49.8)	657	(44.5)	402	(51.3)	634	(44.0)	344	(48.6)	599	(41.3)	321	(47.8)	583	(40.4)	303	(44.6)
Cities (pop 50,000+)	703	(47.1)	387	(42.7)	713	(47.3)	394	(47.0)	752	(51.0)	360	(45.9)	729	(50.6)	336	(47.4)	764	(52.7)	331	(49.2)	781	(54.2)	345	(50.8)
Towns and villages	48	(3.2)	17	(1.9)	76	(5.1)	27	(3.2)	67	(4.5)	22	(2.8)	78	(5.4)	28	(4.0)	87	(6.0)	20	(3.0)	78	(5.4)	31	(4.6)
